# Prevalence, knowledge, attitude, and practice of vaping among university students in the United Arab Emirates: a cross-sectional study

**DOI:** 10.3389/fpubh.2026.1803286

**Published:** 2026-04-28

**Authors:** Zaynab Shuaib Azeez, Layan Al Tawbah, Leen Imad, Enji Zino, Aya Baradie, Sanah Hasan, Jad Matar, Nada M. Kassem, Souheil Hallit, Diana Malaeb

**Affiliations:** 1 College of Pharmacy, Gulf Medical University, Ajman, United Arab Emirates; 2 Department of Clinical Sciences, College of Pharmacy and Health Sciences, Ajman University, Ajman, United Arab Emirates; 3 Center of Medical and Bio-allied Health Sciences, Ajman University, Ajman, United Arab Emirates; 4 The International School of Choueifat, Sharjah, United Arab Emirates; 5 PharmD, School of Pharmacy, Lebanese International University, Beqaa, Lebanon; 6 School of Medicine and Medical Sciences, Holy Spirit University of Kaslik, Jounieh, Lebanon; 7 Applied Science Research Center, Applied Science Private University, Amman, Jordan

**Keywords:** attitude, electronic cigarettes, knowledge, nicotine, practice, students, universities, vaping

## Abstract

**Background:**

Electronic cigarette use is increasing among young adults worldwide, yet data from the Middle East remain limited. Understanding whether vaping behavior is driven more by knowledge or attitudes has implications for prevention strategies. This study aimed to determine the prevalence of vaping among university students in the United Arab Emirates (UAE), assess their knowledge of vaping-related risks, explore attitudes and practices toward e-cigarette use, and identify factors associated with vaping initiation and continuation.

**Methods:**

The study was conducted among university students in the UAE between April 2024 and November 2025. All students aged 18 years and above were eligible to participate. Data were collected using a structured questionnaire incorporating the Electronic Cigarette Knowledge Questionnaire (ECQ), the Electronic Cigarette Attitude Scale (ECAS), and the Penn State Electronic Cigarette Dependence Index (PS-ECDI). Descriptive statistics were used to summarize participant characteristics, while multivariate logistic regression identified factors associated with current e-cigarette use.

**Results:**

Among 422 participants (mean age = 21.5 years ± SD = 4.9; 72.7% female), 99 students (23.5%) reported current e-cigarette use. Knowledge levels were generally low to moderate (mean ECQ score, 8.16 ± 3.62 of 17), with only 3.4% demonstrating high knowledge. Among current users, 51.5% exhibited low nicotine dependence, while 41.5% showed moderate to high dependence. In multivariable analyses, higher positive attitude scores were associated with greater odds of e-cigarette use [adjusted odds ratio (Aor), 1.24; 95% CI, 1.16–1.33; *P* < 0.001], whereas sociodemographic characteristics and knowledge level were not significantly associated with current use.

**Conclusion:**

Vaping is common among UAE university students, with a notable proportion exhibiting nicotine dependence. Targeted educational and campus-based interventions are needed to address misconceptions and reduce vaping-related harms.

## 1 Background

Electronic cigarettes (e-cigarettes) are battery-powered devices that aerosolize nicotine, flavorings, and other chemicals for inhalation (1). Although initially introduced as smoking-cessation aids, they are now widely used for recreational purposes, particularly among adolescents and young adults, driven by appealing flavors, sleek designs, and widespread social and media influence (2).

Since the legalization of e-cigarettes in the United Arab Emirates (UAE) in 2019, availability has expanded rapidly through retail outlets, online platforms (3, 4). While earlier national data suggested a low prevalence of e-cigarette use among adults (5), recent economic projections and observational studies indicate a steady rise in use, especially among the younger age group (6).

Despite growing use, many young adults continue to perceive e-cigarettes as safer than combustible cigarettes, even though aerosol emissions contain toxic substances that pose health risks to users and bystanders (1). Social influence, stress relief, peer norms, and exposure to vaping content on social media have been identified as key drivers of initiation and continued use among young adults in the UAE and other settings (7–9). These factors may reinforce favorable perceptions of vaping that persist even when individuals are aware of potential harms (10, 11).

Prior studies in the UAE and surrounding region have examined vaping prevalence among university students, but gaps remain in understanding how knowledge, attitudes, practices, and nicotine dependence interact to shape behavior (7, 11, 12). Previous studies conducted among university students in the UAE found that the use of e-cigarettes ranges between 6.1% and 23% (13).

The widespread smoking of e-cigarettes in the UAE is influenced by the marketing in high-income countries, favoring the use of electronic nicotine delivery systems and a decline in cigarette smoking (14). Every country has its own regulations regarding e-cigarettes, and these restrictions are constantly changing. The World Health Organization (WHO) has released that electronic nicotine delivery systems require strict regulation to prevent accessibility among youth (15, 16). In the UAE, the sale of tobacco products is forbidden to those under 18 years according to federal law (17).

The objective of this study was to assess the prevalence of e-cigarette use among university students in the UAE and to evaluate their knowledge, attitudes, practices, nicotine dependence, and factors associated with current vaping behavior.

## 2 Method

### 2.1 Study design and location

This was a cross-sectional study conducted among university students in the UAE from April 2024 to November 2025 using snowball sampling. The questionnaire link was shared through social media platforms, including WhatsApp, Facebook, and LinkedIn, and was available for the complete duration of the study. A cover letter that explained the study objectives was included at the beginning of the questionnaire, and the time for the completion of the questionnaire was about 5–10 min.

This study is reported in accordance with the Strengthening the Reporting of Observational Studies in Epidemiology (STROBE) guideline for cross-sectional studies. Similar study designs have been effectively used in previous research across UAE universities to examine comparable topics (7).

### 2.2 Participants

All students enrolled at universities in the UAE who were 18 years of age or older were eligible to be included in the study. This age range was chosen because it's consistent with the legal age for participation and aligns with the approach taken by other regional studies investigating tobacco and e-cigarette use among young adults in the UAE (18).

### 2.3 Survey tools used

The questionnaire had four main sections. The first section collected socio-demographic details like age, gender, academic year, field of study, employment status, family income, and place of living. It also included questions on e-cigarette and cigarette use, including nicotine percentage, preferred flavors, reasons for use, symptoms after use, and promotion of e-cigarettes. The next three sections assessed knowledge, attitude, use, and dependence through various scales.

### 2.4 Knowledge of e-cigarettes

The knowledge score was assessed through the Electronic Cigarette Knowledge Questionnaire (ECQ) (19), an 11-item measure evaluating awareness of e-cigarette constituents, health risks, legality, and general information. Correct responses were scored as 1 and incorrect or “I don't know” responses as 0, with one multi-response item allowing up to 4 points, yielding a total score range of 0 to 17. Higher scores indicated greater knowledge of e-cigarette risks. Knowledge levels were categorized as low (0–7), moderate (8–13), or high (14–17) (12).

### 2.5 Attitudes toward vaping

The attitude score was evaluated using the Electronic Cigarette Attitude Scale (ECAS) (20), a 12-item instrument rated on a 5-point Likert scale. Items were grouped into positive (7 items) and negative (5 items) attitude subscales. Positive items were scored directly, and negative items were reverse-scored, yielding two scores: a positive attitude score (range, 7–35), with higher scores indicating more favorable views, and a negative attitude score (range, 5–25), with higher scores indicating stronger perceptions of harm. Overall attitude was interpreted by comparing the two scores (12).

### 2.6 Usage (practice) and dependence

Students were asked about their e-cigarette usage habits and dependence levels using the Penn State Electronic Cigarette Dependence Index (PS-ECDI) (21), a validated 10-item measure evaluating use frequency, craving, withdrawal symptoms, nocturnal use, and difficulty refraining from vaping. Item scores were summed to yield a total dependence score ranging from 0 to 20, with higher scores indicating greater dependence (12). Dependence levels were categorized as none (0–3), low (4–8), moderate (9–12), or high (≥13) (12).

### 2.7 Reliability of the study scales

The internal consistency of the study scales was assessed using Cronbach's alpha. The knowledge scale demonstrated moderate reliability (α = 0.668). The positive attitude scale showed very good internal consistency (α = 0.898), while the negative attitude scale demonstrated moderate reliability (α = 0.659). The PS-ECDI showed acceptable internal consistency (α = 0.591).

### 2.8 Pilot study and instrument validation

Before the main study was conducted, a small pilot test was conducted with a group of students to ensure that the questions were clear, relevant, and culturally appropriate. Feedback received during this phase helped us make necessary revisions to the questionnaire, improving its clarity and accuracy. This process aligns with best practices in previous UAE research, where pilot studies have been crucial in ensuring that tools are suitable for local use (13).

### 2.9 Ethical considerations

The study was reviewed and approved by the Institutional Review Board at Gulf Medical University, **Ref. no. IRB-COP-STD-39-Sept-2024**. All participants gave their informed consent electronically before answering the questionnaire. Participation was completely voluntary, and all responses were kept anonymous and confidential, in line with ethical standards observed in similar studies within the region (18) and in accordance with the principles of the Declaration of Helsinki.

### 2.10 Sample size calculation

The Epi Info software was used to calculate the minimal sample size required, considering a study power of 80%, a confidence interval of 95%, and a previous prevalence of vaping in the UAE as 39.6% (22). The minimal sample size calculated was 368; this number was increased to allow adequate bivariate and multivariable analyses, and also to take into account refusal rates.

### 2.11 Statistical analysis

The data were analyzed using the Statistical Package for Social Sciences (IBM SPSS Statistics for Windows, Version 25.0; IBM Corp., Armonk, NY, USA). Descriptive statistics summarized participants' socio-demographic characteristics and e-cigarette use patterns. Continuous variables were presented as means and standard deviations, while categorical variables were shown as frequencies and percentages.

Knowledge and dependence scores were categorized into low, moderate, and high levels based on cut-off values, while attitude scores were analyzed continuously, with higher scores indicating more positive or negative attitudes toward e-cigarettes.

Associations between e-cigarette use and independent variables were examined using chi-square tests for categorical variables and independent-samples *t-*tests for continuous variables. Multivariable logistic regression was used to estimate adjusted odds ratios (aORs) with 95% CIs for factors associated with current electronic cigarette use. To avoid multicollinearity, only the positive attitude score from the Electronic Cigarette Attitude Scale was included, while the negative score was excluded. The model adjusted for age, sex, college type, employment status, income, residence, and knowledge category, with statistical significance set at *P* < 0.05.

## 3 Results

### 3.1 Socio-demographic characteristics

A total of 422 university students participated in the study, with a mean (SD) age of 21.52 (4.90) years. Most participants were female (72.7%), undergraduate students (85.8%), and enrolled in medical-related programs (77.7%). Nearly half were in their first year of study (47.2%), and most were unemployed (81.5%). The majority reported living with family or relatives (74.9%) as outlined in Table 1.

**Table 1:** Socio-demographic profile of the study participants (*n* = 422).

Characteristics	Frequency (%)
**Age (Mean** **±SD)**	21.52 (4.9)
Gender	
Female	307 (72.7)
Male	115 (27.3)
Study year	
Year 1	199 (47.2)
Year 2	162 (38.4)
Year 3	61 (14.5)
College	
Medical (pharmacy/medicine/dental…)	328 (77.7)
Non-medical (engineering/computer science/arts....)	94 (22.3)
University level	
Undergraduate	362 (85.8)
Postgraduate	60 (14.2)
Occupation	
Employed	78 (18.5)
Unemployed	344 (81.5)
Family income level	
Low (below 5,000 AED monthly)	50 (11.8)
Middle (5,000 to 20,000 AED monthly)	161 (38.2)
Upper-middle (20,000 to 50,000 AED monthly)	140 (33.2)
High (above 50,000 AED monthly)	71 (16.8)
Place of living	
Own house	53 (12.6)
University dorms	53 (12.6)
With family or relatives	316 (74.9)

### 3.2 Prevalence and patterns of vaping use

**Figure 1:**
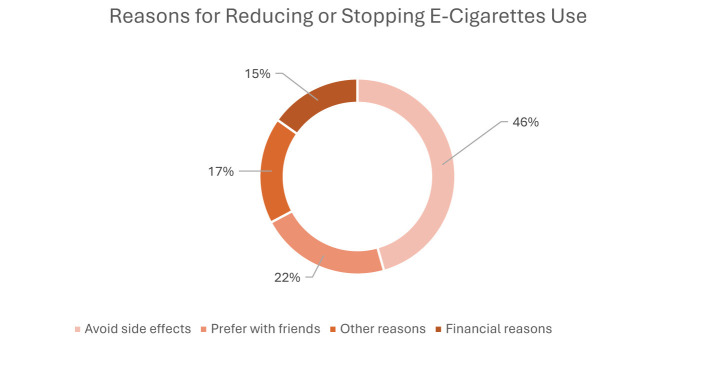
Reasons for reducing or stopping e-cigarette use (*n* = 99).

**Figure 2:**
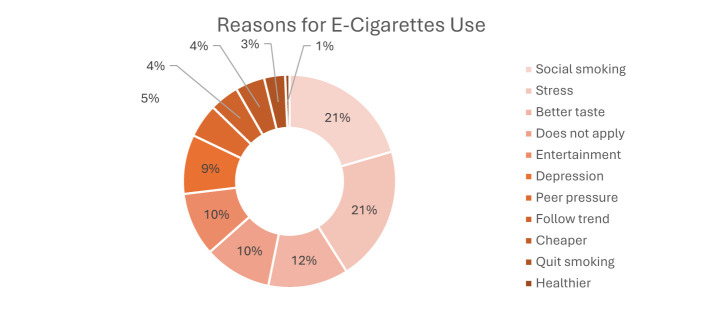
Reasons for e-cigarette use (*n* = 99).

**Figure 3:**
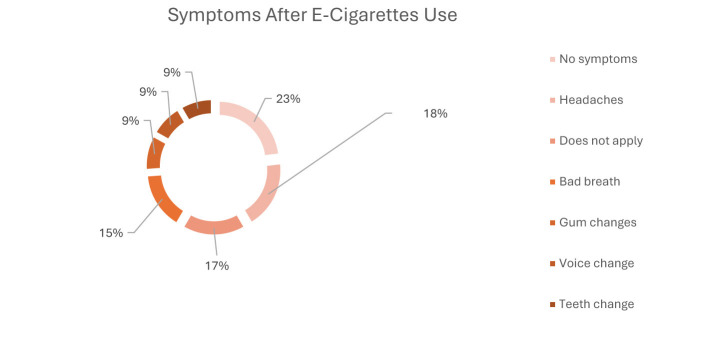
Symptoms after e-cigarette use (*n* = 99).

Most students (77.0%) reported never smoking traditional cigarettes, while 18.5% identified as smokers and 19.0% reported past daily smoking. Regarding e-cigarette use, 68.2% had never vaped, and 23.5% reported current use. Among current e-cigarette users (*n* = 99), 28.3% were unaware of the nicotine concentration used; the most commonly reported strength was 5% (27.3%), and mixed flavors were most frequent (41.4%). Table 2 summarizes the patterns of cigarette and e-cigarette use among students. As shown in Figure 1, the most commonly reported reasons for e-cigarette use were social smoking and stress relief (each 21%), followed by better taste (12%), entertainment (10%), trend-following (4%), and peer pressure (5%) (Figures 1–3). Headaches (18%) and bad breath (15%) were the most frequently reported side effects, although 23% reported no symptoms. The primary reasons for reducing or stopping use were avoiding side effects (46%), social preferences (22%), and financial reasons (15%).

**Table 2:** Overview of cigarette use, vaping patterns, and related behaviors.

Variable	Category	Frequency (%)
**Cigarettes use**	Never	325 (77)
	Daily in the past	19 (4.5)
	Once or twice a month	49 (11.6)
	Once or twice daily	29 (6.9)
**E-cigarette use**	Yes	99 (23.5)
	No	323 (76.5)
**Electronic cigarettes (e-cigarettes) use pattern**	Never	288 (68.2)
	Daily in the past	35 (8.3)
	Once or twice a month	43 (10.2)
	Once or twice daily	56 (13.3)
**Nicotine percentage used among e-cigarette users (*****n*** **=** **99)**	No nicotine	15 (15)
	I don't know	28 (28)
	2%	17 (17)
	3%	5 (5)
	5%	27 (32)
	Doesn't apply	7 (7)
**Flavors used among e-cigarette users (*****n*** **=** **99)**	Coffee flavor	5 (5)
	Mint flavor	5 (5)
	Fruit flavor	32 (32)
	Mixed flavors	41 (41)
	No flavors	10 (10)
	Others	6 (6)
**Would you promote or recommend an e-cigarette? (*****n*** **=** **99)**	No	64 (65)
	I don't know	15 (15)
	Yes	20 (20)

### 3.3 Knowledge of university students

The students surveyed had a limited understanding of e-cigarettes. The mean ECQ (Electronic Cigarette Knowledge Questionnaire) score was 8.16 out of 17 (SD = 3.62), with scores ranging from 0 to 15. According to the ECQ classification system, 36.5% of participants fell within the low-knowledge category, while 60.0% demonstrated only moderate knowledge. Only 3.4% were classified as having high knowledge, indicating that a well-informed understanding of E-Cigarettes was uncommon within this population.

### 3.4 Attitude of participants toward e-cigarette use

The survey results revealed diverse attitudes among university students regarding e-cigarettes, as illustrated in Table 3. Approximately one-third of students perceived e-cigarettes as less harmful than traditional cigarettes, while most recognized their addictive potential (77.8%). Support for allowing vaping in public places was limited, and most participants opposed e-cigarette use among teenagers.

**Table 3:** Attitude toward e-cigarette among university students (*n* = 422).

Statement	Strongly agree	Agree	Neutral	Disagree	Strongly disagree	Mean (±SD)
*Positive attitude*	
E-cigarettes are less harmful than traditional cigarettes.	62 (14.7%)	84 (19.9%)	130 (30.8%)	89 (21.1%)	57 (13.5%)	3.01 (±1.24)
E-cigarettes can help people quit smoking.	45 (10.7%)	116 (27.5%)	133 (31.5%)	85 (20.1%)	43 (10.2%)	3.08 (±1.14)
E-cigarettes should be allowed in public places.	40 (9.5%)	57 (13.5%)	108 (25.6%)	98 (23.2%)	119 (28.2%)	2.53 (±1.29)
Using e-cigarettes is a safe alternative to smoking.	36 (8.5%)	55 (13.0%)	109 (25.8%)	121 (28.7%)	101 (23.9%)	2.54 (±1.23)
Vaping makes people look “cool”.	58 (13.7%)	91 (21.6%)	92 (21.8%)	91 (21.6%)	90 (21.3%)	2.85 (±1.35)
I would try an e-cigarette if offered.	41 (9.7%)	82 (19.4%)	79 (18.7%)	86 (20.4%)	134 (31.8%)	2.55 (±1.36)
E-cigarettes should replace cigarettes.	43 (10.2%)	54 (12.8%)	138 (32.7%)	91 (21.6%)	96 (22.7%)	2.66 (±1.24)
*Negative attitude*	
E-cigarettes are addictive.	183 (43.4%)	145 (34.4%)	63 (14.9%)	25 (5.9%)	6 (1.4%)	1.88 (±0.97)
Teenagers should be allowed to use E-cigarettes.	33 (7.8%)	41 (9.7%)	64 (15.2%)	107 (25.4%)	177 (41.9%)	3.84 (±1.28)
E-cigarette vapor is harmful to bystanders.	125 (29.6%)	137 (32.5%)	112 (26.5%)	32 (7.6%)	16 (3.8%)	2.23 (±1.07)
Vaping should be regulated differently from smoking.	54 (12.8%)	85 (20.1%)	158 (37.4%)	82 (19.4%)	43 (10.2%)	2.94 (±1.15)
Users should be considered non-smokers.	30 (7.1%)	43 (10.2%)	82 (19.4%)	124 (29.4%)	143 (33.9%)	3.73 (±1.23)

For the overall sample, the total positive attitude score (maximum possible score = 35) had a mean of 19.22 (SD = 6.99). The most frequently reported scores were 21 (11.6%), followed by 16 (7.6%) and 27 (3.6%). In comparison, the total score for negative attitudes (maximum possible score of 25) had a mean of 14.62 (SD = 3.72).

### 3.5 Practice behaviors and dependence among current vapers

**Figure 4:**
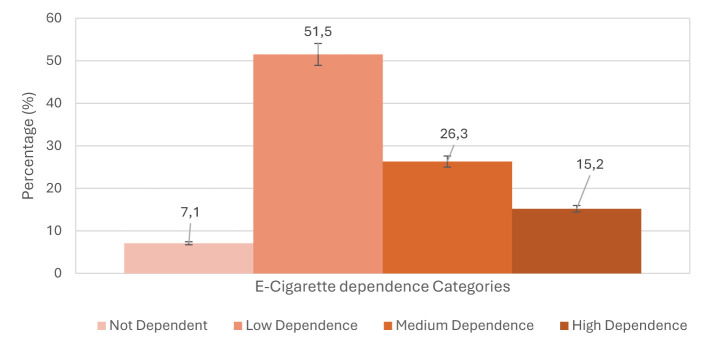
Dependence levels among e-cigarette users (*n*=99) based on the PS-ECDI score.

Among current e-cigarette users (n = 99), the mean PS-ECDI dependence score was 8.22 (SD = 4.06), with scores ranging from 0 to 18. Based on the dependence categories shown in Figure 4, more than half of users (51.5%) had low dependence, 26.3% had moderate dependence, and 15.2% had high dependence. A small proportion (7.1%) showed no evidence of dependence.

### 3.6 Univariate and multivariate analysis

The bivariate analysis showed that demographic and socioeconomic factors, including age, gender, college type, employment status, income level, place of living, and knowledge category, were not significantly associated with e-cigarette use. In contrast, attitudinal factors demonstrated clear associations where current users reported higher positive-attitude scores (*p* < 0.001) and lower negative-attitude scores (*p* = 0.014) than non-users.

Multivariable logistic regression analysis showed that attitudes were the primary factors associated with e-cigarette use. Table 4 summarizes the univariate and multivariable logistic regression analyses. Higher positive attitude scores were associated with increased odds of use [adjusted odds ratio (aOR), 1.24; 95% CI, 1.16–1.33; *P* < 0.001]. None of the sociodemographic variables or knowledge level were significantly associated with electronic cigarette use after adjustment.

**Table 4:** Univariate and multivariate analysis of factors associated with e-cigarette use (*n* = 422).

Variables		E-cigarettes user			Multivariate analysis	
		**No (%)**	**Yes (%)**	***P*** **-value**	**Adjusted OR (95% CI)**	**Adjusted** ***P*****-value**
**Age (Mean** **±SD)**		21.30 ± 4.61	22.24 ± 5.73	0.096	1.04 (0.98–1.10)	0.159
**Gender**	Male	81 (70.4%)	34 (29.6%)	0.070	0.89 (0.49–1.59)	0.682
	Female	242 (78.8%)	65 (21.2%)		1	
**College type**	Medical	257 (78.4%)	71 (21.6%)	0.101	0.60 (0.31–1.15)	0.125
	Non-medical	66 (70.2%)	28 (29.8%)		1	
**Employment**	Employed	59 (75.6%)	19 (24.4%)	0.836	0.55 (0.27–1.14)	0.110
	Unemployed	264 (76.7%)	80 (23.3%)		1	
**Income**	Low	38 (76.0%)	12 (24.0%)	0.252	0.76 (0.30–1.92) (Low vs. High)	0.556
	Medium	128 (79.5%)	33 (20.5%)			
	Upper medium	109 (77.9%)	31 (22.1%)			
	High	48 (67.6%)	23 (32.4%)			
**Place of living**	Own house	38 (71.7%)	15 (28.3%)	0.638	0.99 (0.45–2.18) (Dorms vs. Family)	0.980
	Dorms	40 (75.5%)	13 (24.5%)			
	Family/relatives	245 (77.5%)	71 (22.5%)			
**Knowledge of e-cigarettes**	Low	118 (79.2%)	31 (20.8%)	0.639	0.39 (0.09–1.66) (Low vs. High)	0.201
	Moderate	184 (75.1%)	61 (24.9%)			
	High	11 (78.6%)	3 (21.4%)			
**E-cigarettes positive attitude score**		18.09 ± 6.94	22.91 ± 5.78	**< 0.001**	1.24 (1.16–1.33)	**< 0.001**

SD, Standard Deviation; Adjusted OR, adjusted Odds Ratio; CI, confidence Intervals; Adjusted *P*-value, adjusted *P*-Value.

## 4 Discussion

In this cross-sectional study of university students in the United Arab Emirates, nearly one in four participants reported current e-cigarette use, and more than 40% of users exhibited moderate to high nicotine dependence. Although knowledge of e-cigarette risks was generally low to moderate, knowledge level was not independently associated with vaping behavior. In contrast, favorable attitudes toward e-cigarettes were strongly associated with current use after adjustment for sociodemographic factors, suggesting that attitudes may mediate the influence of social exposure, peer norms, and stress-related motivations on vaping behavior.

### 4.1 Prevalence of use of vape

The prevalence of e-cigarette use in this study (23.5%) is consistent with findings from several Middle Eastern studies, including Qatar (23), Saudi Arabia (24), Palestine (25), and Iran (26), where reported rates among university students range between 15% to 30%. In contrast, studies from Western countries such as the United Kingdom (8), the United States (27), Canada (28), and France (29) have reported higher prevalence rates, often exceeding 30%. These differences may be explained by longer market availability, greater social acceptance, and more intensive marketing strategies in Western settings, as well as cultural and regulatory variations across regions (30).

Consistent with prior research, social smoking and stress relief were the most frequently reported reasons for e-cigarette use among students in this study (8, 23, 24, 27). These motivations suggest that vaping among university students is often driven by social and emotional factors rather than by deliberate smoking cessation. The discreet nature of e-cigarettes, their reduced odor, and their ability to be used in settings where smoking is restricted may further enhance their appeal (8, 31, 32).

### 4.2 Knowledge alone does not provide protection

Knowledge regarding e-cigarettes was limited among participants, with most students demonstrating low to moderate awareness of vaping-related health risks. Similar knowledge gaps have been reported among university students in the Middle East and Asia (25, 33, 34). Knowledge gaps may reflect reliance on social media and marketing rather than reliable health sources, as well as limited educational coverage of e-cigarettes, even among medical students (8, 28). Additionally, vaping is a relatively new behavior with poorly understood long-term health effects, and limited inclusion of e-cigarette education in academic curricula may contribute to knowledge gaps, even among medical students (14).

However, knowledge level was not associated with e-cigarette use in the adjusted analysis. This finding suggests that awareness of harm alone may be insufficient to deter vaping. Even when students recognize that e-cigarettes may be harmful or addictive, favorable perceptions, stress-related motivations, and peer norms may override cognitive risk appraisal.

### 4.3 Attitude is the primary driver

The results of this study reveal that university students hold mixed and sometimes contradictory attitudes toward e-cigarettes. While many students recognize that e-cigarettes can be addictive and harmful, a significant number still believe that vaping is safer or less harmful than conventional cigarettes. This combination of concern and reassurance indicates uncertainty about the true risks of vaping. Similar patterns have been observed among university students in various Middle Eastern countries, where awareness of the potential harms coexists with favorable perceptions of e-cigarettes (23, 25, 33).

This divergence between knowledge and behavior reflects patterns commonly observed in health behavior research. Behavioral decisions are often shaped not only by factual knowledge but also by attitudes, perceived social norms, and emotional motivations. Favorable perceptions of vaping, normalization of the behavior within peer networks, and beliefs that vaping can relieve stress may encourage continued use even among individuals who are aware of potential risks. These dynamics suggest that interventions aimed solely at increasing awareness of health risks may have limited impact unless they also address the underlying attitudes and social influences that sustain vaping behavior (34, 35).

### 4.4 Practice related to vaping

The findings of this study indicate that e-cigarette use among university students is often regular rather than occasional. More than half of current users exhibited low dependence, while a significant portion displayed moderate (26.3%) to high dependence (15.2%), highlighting a real risk of nicotine addiction. This suggests that vaping among students frequently goes beyond social or experimental use and can evolve into more habitual behavior over time.

These patterns of dependence may be explained by the vaping practices and motivations of students. Many users reported frequent or daily use and were unaware of the nicotine concentrations in the products they consumed, which could lead to unintentional high nicotine intake. Additionally, vaping was commonly used in social settings or as a way to cope with stress, reinforcing repeated use and increasing the risk of dependence. The widespread appeal of flavored e-cigarettes may further encourage continued use by making vaping more enjoyable and reducing concerns about its harmful effects. Similar patterns of regular use, limited awareness of nicotine content, and moderate to high dependence have been observed among university students in several Middle Eastern countries, suggesting that this issue is a regional concern rather than an isolated finding (23, 24, 34).

Together, these findings indicate that e-cigarette dependence among university students is driven by a combination of behavioral habits, nicotine exposure, and positive perceptions of vaping, rather than intentional addiction. This highlights the importance of addressing vaping practices and underlying motivations, alongside awareness of health risks, when developing interventions aimed at reducing nicotine dependence in this population.

### 4.5 Limitations and strengths

This study has several strengths, including a relatively large sample size, the inclusion of students from multiple academic programs, and the use of validated instruments to assess knowledge, attitudes, and nicotine dependence. The simultaneous examination of prevalence, perceptions, behaviors, and dependence provides a more comprehensive understanding of vaping among university students than studies focusing on single dimensions. Additionally, the use of anonymous, self-administered questionnaires likely reduced social desirability bias and encouraged honest reporting.

Despite its strengths, several limitations must be acknowledged. The cross-sectional design prevents establishing causality between knowledge, attitudes, and vaping behavior. Although associations can be identified, temporal relationships or changes over time cannot be inferred. The study relied entirely on self-reported data, which may be subject to recall bias, underreporting, or overreporting, particularly for sensitive behaviors such as nicotine use, dependence symptoms, or reasons for vaping. The sample was obtained mainly from two universities in a specific region of the UAE, where most of the participants were medical students. Although this reflects the institution's student demographics, it may limit the generalizability to students in other universities in another region or country, especially those with more diverse academic disciplines or different socio-economic backgrounds. Another limitation is that some scales, although validated internationally, have had limited prior cultural adaptation in the UAE. While a pilot study was conducted, certain items may still differ in interpretation across cultural or linguistic contexts. Although the study captured some elements of environmental and policy-level factors such as exposure to information sources (eg., social media, advertisements) and knowledge of national regulations on e-cigarette scales and use, it did not comprehensively assess contextual influences such as campus-specific vaping regulations, enforcement practices, ease of access to vaping products, or the intensity and frequency of exposure to social media marketing. These unmeasured factors may influence vaping initiation and continuation among students and should be explored in future research. Additionally, dependence was assessed only among current e-cigarette users, resulting in a smaller subsample (n-99), which may reduce the statistical power of the dependence-related analysis. Finally, because the survey was completed online, students without regular access to digital platforms may have been unintentionally excluded.

### 4.6 Study implications

The finding that attitudes, rather than knowledge, were the primary correlates of vaping suggests that interventions should target the perceptual and social pathways that shape nicotine use. The relatively high prevalence of e-cigarette use, combined with the strong association between attitudes and vaping behavior, suggests that interventions addressing perceptions, social norms, and stress-related motivations may be more effective than approaches based solely on risk education (7, 12). Prevention strategies informed by these data would likely require modification of perceptions, social cues, and affective motivations in addition to providing information about health risks (9, 36–38), unless they also address perceptions, social norms, and motivations such as stress relief and peer influences (8).

The presence of knowledge gaps even among students in health-related fields supports the need to integrate up-to-date education on e-cigarettes and nicotine dependence into academic curricula (11, 12, 18).

At the policy level, the study's findings support the need to strengthen institutional and governmental regulations regarding access to and use of e-cigarettes. Universities may consider adopting clearer policies on vaping within campus premises, increasing enforcement of smoke-free/vape-free zones, and offering cessation support programs tailored to young adults (39). Given that a significant proportion of users reported medium to high dependence, universities should collaborate with student health services to establish screening, counseling, and cessation support tailored to young adults (40).Finally, at the broader public health level, the findings highlight the need for continued monitoring of vaping trends among young people in the UAE. Given the evolving nature of e-cigarette products and making strategies, ongoing surveillance can help policymakers stay informed about emerging risks and adapt regulations accordingly (10, 11).

## 5 Conclusion

E-cigarette use appears to be relatively common among university students, with approximately one in four reporting current use. Although gaps in knowledge about e-cigarettes remain evident among many students, the results suggest that knowledge alone does not play a decisive role in shaping vaping behavior. Rather, attitudes toward vaping emerge as a more influential factor. Students who view e-cigarettes as less harmful, socially acceptable, or useful for coping with stress are more likely to engage in vaping, even when they possess some awareness of potential health risks. This indicates that perceptions and social contexts may outweigh factual understanding in guiding behavioral choices related to vaping.

Furthermore, the findings suggest that vaping among students may extend beyond casual experimentation. A notable proportion of users exhibit signs consistent with nicotine dependence, highlighting the potential for regular use to develop relatively quickly. This pattern raises concerns about the normalization of vaping within university environments and the risk of sustained nicotine exposure among young adults.

Taken together, these findings highlight the need for prevention approaches that extend beyond knowledge-based education. Efforts to reduce vaping among university students may be more effective when they address the underlying attitudes, social influences, and coping behaviors that support vaping practices. Strengthening campus-level policies and providing accessible support for students experiencing nicotine dependence may also contribute to reducing long-term health risks associated with e-cigarette use.

## Data Availability

The original contributions presented in the study are included in the article/supplementary material, further inquiries can be directed to the corresponding author.
